# Two novel prehabilitation apps to help patients stop smoking and risky drinking prior to hip and knee arthroplasty

**DOI:** 10.1007/s00264-023-05890-y

**Published:** 2023-08-08

**Authors:** Hanne Tønnesen, Rie Raffing, Susanne Vahr Lauridsen, Jes Bruun Lauritzen, Anne Marie Halmø Elholm, Helle Sæderup Jensen, Peter Espinosa, Karl Åke Jansson, Anne H. Berman, Jenaro Fernández-Valencia, Ernesto Muñoz-Mahamud, Manuel Santiñà, Andrés Combalia

**Affiliations:** 1 WHO CC (DK-62), Clinical Health Promotion Centre, The Parker Institute, Bispebjerg & Frederiksberg Hospital, University of Copenhagen, Copenhagen, Denmark; 2 Department of Orthopedic Surgery, Bispebjerg & Frederiksberg Hospital, University of Copenhagen, Copenhagen, Denmark; 3 Department of Molecular Medicine and Surgery, Karolinska Institute at Reconstructive Orthopaedic Surgery, Karolinska University Hospital, Stockholm, Sweden; 4 Department of Clinical Neuroscience, Karolinska Institute, Stockholm & Department of Psychology, Uppsala University, Uppsala, Sweden; 5 Department of Orthopedic Surgery, Hospital Clinic Barcelona and Faculty of Medicine & Health Sciences, University of Barcelona, Barcelona, Spain

**Keywords:** Perioperative risk reduction, Digital lifestyle intervention, Alcohol, Tobacco, Apps

## Abstract

**Purpose:**

Daily smoking or risky drinking increases the risk of complications after surgery by ~50%. Intensive prehabilitation aimed at complete cessation reduces the complication rate but is time-consuming. The purpose of this study was to carry out preoperative pilot tests (randomized design) of the feasibility (1A) and validation (1B) of two novel prehabilitation apps, habeat® (Ha-app) or rehaviour® (Re-app).

**Methods:**

Patients scheduled for hip or knee arthroplasty with daily smoking, risky drinking, or both were randomised to one of the two apps. In part 1A, eight patients and their staff measured feasibility on a visual analog scale (VAS) and were interviewed about what worked well and the challenges requiring improvement. In part 1B, seven patients and their staff tested the improved apps for up to two weeks before validating the understanding, usability, coverage, and empowerment on a VAS and being interviewed.

**Results:**

In 1A, all patients and staff returned scores of ≥5 for understanding the apps and mostly suggested technical improvements. In 1B, the scores varied widely for both apps, with no consensus achieved. Two of four patients (Ha-app) and one-third of the patients (Re-app) found the apps helpful for reducing smoking, but without successful quitting. The staff experienced low app competencies among patients and high time consumption. Specifically, patients most often needed help for the Ha-app, and the staff most often for Re-app; however, the staff reported the Re-app dashboard was more user-friendly. Support and follow-up from an addiction specialist staff member were suggested to complement the apps, thereby increasing the time consumption for staff.

**Conclusions:**

This pilot study to test prototype apps generated helpful feedback for the app developers. Based on the patient and staff comments, multiple improvements in functionality seem required before scaling up the evaluation for effect on prehabilitation and postoperative complications.

## Introduction

Unhealthy lifestyle behaviors are important and preventable risk factors for patients undergoing surgery. Among these behaviours, smoking and risky drinking add about 50% to the classic risk factors for developing complications [1, 2]. Recently, daily smoking and social alcohol drinking have been included in the American Society of Anesthesiology (ASA) international preoperative risk evaluation (the ASA score). Thus, a healthy patient in the lowest risk group is now defined as “healthy, non-smoking, no or minimal alcohol” [3].

Patient groups with smoking and risky drinking develop the same type of complications as all other patients, but they develop them much more frequently [1, 2, 4, 5]. The pathophysiology behind the increased risk involves the suppression of multiple organ systems [6]. Organ dysfunction also exists in patients without alcohol-induced liver disease or smoking-induced lung disease [6]. The organ damage is often subclinical and occurs at the cellular level, thus reducing the extra capacity that usually supports the patient during surgery and recovery. The result is a significantly increased risk of postoperative complications. However, complete abstinence from smoking and drinking for one to two months leads to sufficient recovery of organ functions, thereby reducing the complication rate [7].

Recently, strong evidence has shown significant risk reduction (leading to an approximate halving of postoperative complications after surgery) following patient participation in a four to eight week intensive program aiming at the complete cessation of smoking and drinking before surgery [8, 9]. This is also the case for arthroplasties, which have been the subject of several randomized trials on perioperative smoking and alcohol intervention [10–12]. In contrast, shorter intervention programs do not affect postoperative complications [8, 9, 13]. Likewise, there is no evidence of an effect on complications of only reducing the amount of smoking and intake preoperatively rather than ensuring complete cessation [12].

The intensive programs for preoperative cessation of smoking and alcohol consumption include pharmacological support, patient education, and motivational support. They are followed by high quit rates for smoking and alcohol and a significant reduction in complication rates [8, 9]. Implementing new evidence in the clinical setting is often slow and fragmented [14] and could benefit from effective modern communication and new technology.

A recent systematic review of perioperative digital behavior change interventions identified only one full-scale randomized trial [15], performed by Lemanu and colleagues on 2 × 44 patients [16]. They evaluated the efficacy of a daily text message for four to six weeks preoperatively to encourage physical activity concerning bariatric surgery. However, significantly better perioperative adherence to exercise advice in the group receiving text messages was not followed by a change in the distance walked during the six minute walk test or in postoperative complications [16]. More randomized trials are required. An important step toward that would be to pilot-test two newly developed apps and related dashboards aimed at prehabilitation involving successfully quitting smoking and risky drinking prior to surgery.

Here, we evaluate the functionality of these two apps in terms of the feasibility of the IT solutions (IA) and as validation regarding understanding, applicability, and support (IB) of the two new apps by the patients and their staff in the clinical setting.

For IA, the hypothesis was that at least one of the prototypes would score at least 5 on a visual analog scale for feasibility. For IB, we hypothesized that a substantial agreement (i.e., Kappa > 0.6) [17] on the validation of the improved prototypes would be obtained among the patient groups and the staff members.

## Materials and methods

This H2020-supported project was initiated by researchers at the surgical and IT departments at three European university hospitals.

### Pre-clinical steps

Before this clinical pilot on evaluation of the two IT solutions, development and selection processes regarding app development were carried out. This included two earlier phases with sprint tests, reports, and approval by the project group. These first steps were completed before the introduction to the clinical setting; in this last phase, the two most promising IT solutions of the original 15 proposals were further developed to be relevant for the clinical setting.

### Hierarchical design

We used a hierarchical design for this randomized pilot, beginning with the first part (IA) and allowing for some last adjustments before the next pilot part IB:

- IB would begin only if IA was completed successfully (i.e., the prototype worked well in the clinical set-up as measured by a score of at least 5 on a 0–10 visual analog scale)
- A final and sizable randomized trial of the efficacy on lifestyle behaviors and postoperative complications would be triggered only if a substantial agreement was achieved among the patient and the staff groups in IB (defined as Kappa above 0.6 for understanding, usability, covering, and managing lifestyle improvement).

Risky drinking was defined as an intake ranging from above 14 drinks per week or at least 25 g ethanol per day on average. Daily smoking was defined as any everyday tobacco smoking, with or without dependence symptoms.

### Criteria and recruitment

The recruitment for convenience samples took place from April 2020 to September 2021 at the orthopaedic departments with stratification at the national level and with blocks of unknown size (ranging from 1 to 3). Recruitment was closed due to the lockdown of elective surgery by the COVID-19 pandemic. For the feasibility test (IA), eight patients fulfilling the inclusion criteria (and meeting none of the exclusion criteria) were included with their six staff members. However, in case of major app adjustments, it would be necessary to repeat the 1A part and include more participants. For the validation (1B), we aimed to include ten other patients for the apps in each country; altogether, 30 patients and their staff members.

All staff had two to four of training sessions each per app. The inclusion criteria were adult patients scheduled for hip or knee arthroplasty with daily smoking, risky drinking, or both. Exclusion criteria were not being able to give informed consent (e.g., being less than 18 years old, having a severe mental illness, not understanding the national language), acute surgery, withdrawal of consent, being pregnant or lactating, not having or being able to press the buttons, or otherwise use a smartphone/tablet.

### The IT solutions

The evaluation included two apps with related dashboards and the working titles Habeats® (Ha-app) from 8Wires® and Rehavior® (Re-app) from Eurecat®, both aiming to support patients quit daily smoking and risky drinking in the perioperative period, thereby reducing the risk of complications. The apps and dashboards were not connected to the medical record systems, as they used cloud solutions for data storage, which is not allowed for safety reasons; thus, anonymous data collection at the source was used for the IT solutions (see below).

### The intervention

The IT intervention in the Re-app was based on the above-described, highly effective face-to-face intensive smoking and alcohol cessation interventions, which also inspired the Ha-app [8, 9]. The intensive interventions include an education program for the patient, motivational support, feedback tools, and recommendations for supportive medicine (over the counter), such as nicotine replacement therapy, based on individual dependency measurements and preferences. Medication was not part of the apps. Both apps used artificial intelligence with videos or charts for information, recommendations, intervention, and praising of good results in patient feedback. During the project, a daytime hotline to the project staff was available to the participants.

### Data collection and handling

Data were stored at the secure server for sensitive data in Region H. After informed consent, the clinical project group collected data on lifestyle behaviours and changes via interviews, the apps, and the dashboard. Data on the date and type of surgery were collected from the medical records. The IT companies extracted data on the duration of app use, the number of visits, and the opening and completion of games/chares.

The data handling conformed to the GDPR agreement. There was no contact between IT companies and patients. The project staff had contact with each IT company and did not share any personally identifiable information. Only the clinical project group had access to personally identifiable data.

Due to the cloud storage of data, there was no personally identifiable information in the apps or the dashboard, and the IT companies had no access to personally identifiable data. Anonymous pre-paid cards or smartphones were handed out. Only a random number or nickname was used in the app and dashboard, thereby making it visible to the IT companies.

### Statistics

In IA, the feasibility was documented by describing the process from the log registration and response using a VAS with a cut-off value of 5. The time spent on the test was compared between the groups by a Mann-Whitney test; *p* < 0.05 was considered significant.

In IB, using Kappa statistics, the validity was analyzed for inter-variability among patients and staff. A substantial agreement was considered acceptable, i.e., a result of Kappa > 0.60. However, 0.81–1.00 would be considered an almost perfect agreement.

The pilot results were expected to be used for the following power calculation regarding a possible final randomized trial on efficacy (part II).

### Ethics, risks, side effects, and inconvenience

All participants were included after informed consent and could withdraw their consent and decide to make their data deleted at any time without further explanation and with no impact on the treatment and care in the department. The Scientific Ethical Committee (VEK-number H-21018467) in Denmark and the local hospital-based IRB approved the pilot and the final project before starting.

Inclusion in the project involved few and low risks, mainly originating from the collection and storage of sensitive data for patients, which was lowered even further by carefully following the guidelines for each procedure. In addition, changing lifestyle behaviours could be temporarily uncomfortable for patients, but the possible effect might balance this over time of reduced postoperative complications and better health.

The participants had access to a hotline (e-mail or phone) for the clinical project staff during the weekday. The participants were free to use other healthcare services to support smoking and alcohol cessation.

## Results

Overall, the randomized pilot was doable for evaluating the app prototypes. It collected useful data on what worked well and identified several problems and challenges requiring solutions. Both apps required improvement during the feasibility test (1A) to prepare for the following validation (1B). There were no patients lost during the intervention and follow-up.

**Fig. 1:**
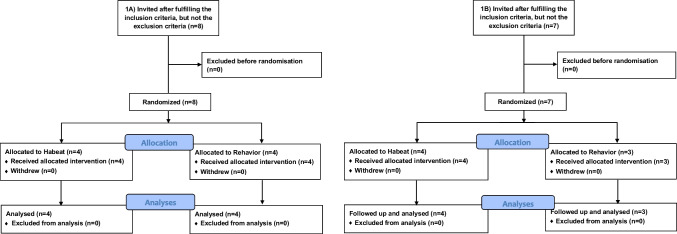
CONSORT flow diagram for the feasibility (1A) and the validity test (1B)

The project and, thereby, the patient recruitment were canceled from 2 April 2020 due to hospital reorganization to meet the acute needs during the COVID-19 pandemic. Afterward, elective surgery was only partly re-opened. However, part IA has been completed with the inclusion of eight patients and their staff, but only seven patients were recruited for part IB; see the consort diagram in Fig. 1 and characteristics in Table 1. None of the included patients were lost during the intervention and follow-up.

**Table 1:** Characteristics of the eight patients in pilot 1A and the seven other patients in the 1B validity test, given as numbers (%) and median (range)

	1A Ha-app (*n* = 4)	1A Re-app (*n* = 4)	1B Ha-app (*n* = 4)	1B Re-app (*n* = 3)
Men	2 (50%)	2 (50%)	2 (50%)	2 (67%)
Age (years)	73 (47–80)	65 (37–81)	56 (54–69)	51 (47–57)
Knee prosthesis	3 (75%)	1 (25%)	0	1 (33%)
Hip prosthesis	1 (25%)	3 (75%)	4 (100%)	2 (67%)
*Lifestyle*				
Daily smoking	3 (75%)^1^	3 (75%)	4 (100%)	3 (100%)
Cigarettes/day	20 (4–30)	10 (8–18)	24 (10–30)	8 (5–20)
Time from wake-up to smoking (min)	6 (6–60)	6 (6–60)	17 (6–30)	30 (6–>60)
Risky alcohol drinking	2 (50%)^1^	1 (25%)	0	0
Drinks/week	18 (15–21)	42 (42–42)	-	-
*Technical information*				
Android smartphone	3 (75%)	2 (50%)	3 (75%)	2 (67%)
iPhone	1 (25%)	2 (50%)	1 (25%)	1 (33%)
Saving of data	Cloud	Cloud	Cloud	Cloud

^1^One participant had both

### Feasibility

The evaluation in IA showed similar scores for both Ha-app and Re-app among patients and staff, respectively (Table 2). This was also the case for several comments regarding what worked well and identifying challenges and problems. Most of the challenges and problems during IA were solved by the patients themselves and the staff, but some required help from the IT companies to be solved. The clinical time consumption was relatively high, but significantly lower for the Re-app (Table 2).

**Table 2:** Functionality (1A) and validation (1B) of the apps, given as median (range)

1A	Ha-app (*n* = 4)	Re-app (*n* = 4)
*Patient responses to the apps*		
After you have opened the app and put in your data, how well do you think it works on a VAS scale from 0 to 10 (0 = not at all; 10 = fantastic good)?	7.5 (2.4^2^–10.0)	9.0 (5.0–10.0)
After solving (or discussing) problems or challenges, how well do you think the app works now on the VAS scale?	7.5 (2.3^2^–10.0)	9.0 (5.0–10.0)
Time spent on the pilot test (minutes)	45 (30–49)*	30 (20–36)
Number of visits to the app	14 (3–46)	2 (0–5)
The total duration of the visits	27 (8–31)	22 (0–33)
*Staff responses*		
After introducing the app to one or more patients, how well do you think it works on the VAS scale?	6.5 (4.7–10.0)	7.5 (6.5–10.0)
After solving (or discussing) problems or challenges, how well do you think the app works now on the VAS scale?	7.0 (6.0–10.0)	8.5 (7.0–10.0)
*Patient responses to the apps*		
Do you understand how to use the app on a VAS scale from 0 to 10 (0 = not at all; 10 = fantastic good)?	9.0 (5.0–10.0)	10.0 (10.0–10.0)
Do you think the app can be used to change your personal lifestyle on the VAS scale?	5.5 (3.0–10.0)	3.0 (2.0–8.0)
Does the app cover your personal situation on the VAS scale?	5.5 (3.0–10.0)	3.0 (2.0–10.0)
Do you feel the app makes you capable of taking control over the lifestyle change on the VAS scale?	7.0 (4.0–10.0)	5.5 (3.0–8.0)
Time spent on the interview (minutes)	7 (7–10)	10 (7–10)
Number of visits to the app	-	22 (8–35)
The total duration of the visits	-	47 (21–73)
Number of clicks to the app (minutes)	114 (0–182)	-
The total duration of clicks (minutes)	122 (0–216)	-
Tasks/charts and videos completed	No data yet	22% (3–40)
*Staff responses:*		
Do you understand how to use the app on the VAS scale?	10	10
Do you understand how to use the dashboard on the VAS scale?	7	9
Do you think the app can be used for your patient group on the VAS scale?	7	7
Does the app cover your patient group on the VAS scale?	7	7
Does the app make your patients capable of taking control over the lifestyle change themselves on the VAS scale?	5	5

^2^One participant being risky drinking identified stigmatizing language, which was not solved by the IT company

*P = 0.05

Only one patient in 1A scored lower than 5 on the pre- and post-improvement tests. The patient felt the language stigmatizing concerning the risk of drinking in the Ha-app and scored 2.4 on the VAS at the test. Several other problems were mentioned, which were well solved, but not the language challenge, and the patient still scored below the value 5, which was the lower limit for the app to continue to part 1B (Table 2). However, another patient reporting risky drinking did not comment upon the language in that app.

Considering the small numbers in the pilot, there seemed to be a tendency for the Re-app to require a shorter time for testing the functions, showing fewer problems and challenges for the patients, and managing to develop solutions for all challenges and problems within the test period compared to the Ha-app (Tables 2 and 3).

**Table 3:** Patients and staff comments (in comprised form) on the functionality of the two tested apps, and problems and the groups involved in the solutions (a: solved by patients; b: solved by project staff; c: solved by the IT company; d: not solved) as well as the staff comments on the dashboard functionality (1A and 1B)

1A – patient comments		Staff comments	
Ha-app	Re-app	Ha-app	Re-app
*What went well?*			
2 × all	All	All	All
Overview	Use and overview	Navigation	Navigation
Download	Download	Download	Installation and use
Mindfulness	Introduction		
Questions	2 **×** questions		
	Password choice		
	Text size		
*What were the problems and challenges – and who solved them? Patients=a; Project staff=b; IT Companies=c; Not solved=d *			
App experience necessary^a+b^	App experience necessary^a+b^	App experience necessary^a+b^	App experience necessary^a+b^
Answers required before program start^a+b+c^	Logging in with e-mail^b^	Answers required before program start^a+b+c^	Answers required before program start^a+b+c^
3 × calendar^a+b^	Reading (small text)^a^	Authenticator^c^	Instruction by phone^b^
Hospital choice^a^	Reading (low contrast)^a^	Not intuitive^d^	Reading (small text)^a^
Icon understanding^a+c^			Hitting buttons^a^
Scroll down^a+c^			
Language intimidating (alc)^d^			
App freezing^a^			
Staff comments to the dashboard functionality			
Ha-app		Re-app	
*What went well?*			
Registration		Worked well	
Installation		No difficulties	
*What were the problems and challenges?*			
Difficult due to hospital antivirus protection (firewall)		Frequent use necessary to remember the functions	
		Many questions before program start	
		Lost patient in dashboard	
1B – patient comments			
*What went well?*			
The app helps to lower tobacco use		Activities are simple and quick to do and help to reduce tobacco use	
Understands the app quickly			
Lowers tobacco consumption; smoke in designated areas			
*What were the problems and challenges?*			
The job does not allow to spend time using the app. Uncertain if it supports lifestyle change		Initial app accessing difficulties made the patient leave. Prefers to change lifestyle without the app	
Does not expect to stop smoking because it helps to endure pain			
Forget to use the app at work. Messages do not motivate, and questions are too simple.			
Messages and explanations. The app does not propose things except for mindfulness and that doesn’t motivate. Works a lot and does not have time to use the app			

### Validation

After improvements, the new versions were evaluated among seven patients who were daily smokers (Fig. 1) and their staff members. Overall, feasibility evaluation was possible using the VAS score for all four outcomes for patients and five for staff.

From a clinical point of view, the required level of involvement for solution of problems identified during the study is of importance; i.e. patients, staff and/or the company. All patients and staff scored 5 or more regarding understanding how to use the two improved prototypes, but the scores varied widely for both apps, and no substantial agreements were obtained (Tables 2 and 3). In total, three expressed that the app helped them to reduce smoking, 2/4 for the Ha-app and 1/3 for the Re-app. No patients had successfully quit smoking during the test.

The staff VAS scores and comments were rather similar for both apps. Overall, the challenges and problems experienced were mainly that the patients had minimal experience with using apps and did not have the necessary time to use the apps due to work reasons. The staff found the Re-app-related dashboard the most user-friendly.

Both apps were time-consuming: the Ha-app for the patients who often needed help and the Re-app for the clinical staff. The staff recommended support and follow-up from an addiction specialist as complementary to the apps.

## Discussion

This study showed that the feasibility test identified several technical challenges requiring help from patients, staff, and IT companies. The time spent on the feasibility test was the longest for the Ha-app despite training workshops for the staff. The validity test results showed no substantial agreement for any of the apps. Some reduction in the number of cigarettes was reported in both apps after testing them for up to two weeks, although no participants managed to quit.

Recruitment, continuity, and time consumption are general challenges in digital behaviour change intervention studies concerning surgery [15]. In this study, the time consumption for introduction and use and clinical involvement is worth considering concerning the intensive face-to-face interventions, which have been proven effective regarding both lifestyle behaviour changes and consequent reduction of complications. These intensive interventions are defined by four face-to-face sessions of ten minutes each or more, psycho-social and health education, pharmacotherapy, and motivational support individualized to personal needs regarding health literacy and other conditions of importance for the outcomes [18].

Despite a great potential for using effective IT solutions for perioperative lifestyle behavior risk reduction via lifestyle interventions, at the time of this study only four products have been evaluated in randomized trials: three pilot studies and one full-scale trial. Another six feasibility studies have been performed with only one arm and mainly using text messages. As most have used convenience samples, it is not surprising that the patient groups, in general, were positive. In addition, many of those, similar to this study, showed a need for optimizing functionality, IT literacy requirements, and time consumption reduction [19–24].

In addition to the earlier mentioned randomized trial by Lemanu and colleagues [16], three pilot studies have been performed in a randomized design [25–27]. They included 145 patients in total, and all reported some tendencies toward effects on lifestyle improvement. DeMartini and colleagues evaluated the effect of text messages aiming at alcohol abstinence concerning liver transplantation. At the eight week follow-up, zero of eight in the intervention group compared to two of six in the standard care group had positive bio-markers indicating alcohol intake [25]. Krebs and colleagues used gamification for an intervention aiming at smoking abstinence concerning lung and gastrointestinal cancer surgery. After one month, the confirmed abstinence was four of 13 versus two of 11 [26]. Van der Velde and colleagues tested a smartphone app 14 days preoperatively for 86 patients undergoing major surgery. Only 11 patients were frequent alcohol users, and seven were smokers. The self-reported results showed that two of two vs. one of nine patients reduced their alcohol intake, three of the seven smokers were followed up, and one in each group had reduced or successfully quit smoking [27].

Further to a possible positive tendency, the sparse randomized studies hitherto indicate that objectively measurable outcomes or other validations are necessary in addition to self-reported effects.

### Strengths and limitations

It was a strength that the estimated number of patients was included in part (IA), but the low number of participants biased the validation part (IB), and only smokers participated. The preliminary results of IB are probably not representative of the apps. Furthermore, the recruitment was strongly impacted by the COVID-19 pandemic, and participants were not included consecutively, which could lead to a selection bias.

Self-reported alcohol intake may be underestimated, leading to false negative responses. In contrast, it is not over-estimated. For smoking, the under-estimation is lesser, and also, here, over-estimation is rather seldom. Overall, this means that if the patient report to be a smoker or drinking risky, as in this project, the risk of a false positive seems neglectable.

The preliminary prototypes evaluated in IA were improved during the test period, and therefore, the final versions tested in IB differed from those in IA; thus, the results and comments cannot be compared across IA and IB. For security and safety reasons, the apps and the dashboards in this study were not connected to the department’s patient recording system. This may have increased the already high need for staff resources and competencies to introduce the apps to the patients, use the dashboard, and follow up, all of which may be a limitation. In general, pharmacotherapy is recommended to increase the successful quit rate in the perioperative period [13], but this was not part of the intervention integrated into the apps, which could bias the results and lower the usefulness in the clinical setting.

The length of education and level of health literacy of the patients may impact the compliance with programs aiming at lifestyle intervention. However, in previous face-to-face studies, the intensive programs for lifestyle intervention used in this project have an overall major effect on smoking and drinking across the patient groups [6, 8, 9]. All the patients received similar information before inclusion. In addition, they received the educational material handed out as part of the clinical routines.

This study should be generalized only with caution beyond Europe. Even across Europe, the cultural differences include differences in IT literacy and language. Thus, phrases may be stigmatizing by some persons and not by others; this could even be the case within the same country or municipality. Language may be considered more stigmatizing in an app without staff to adapt a dialog to the patients’ different vulnerabilities and situations. This could limit outreach via apps and other IT solutions to surgical patients needing perioperative risk reduction.

### Perspectives

In contrast to the seldom performed effect studies on publicly accessible apps targeting lifestyle improvement, increasing evidence has been gathered for apps aiming at disease management, early identification of exacerbations, and symptom control for non-communicable diseases. This should also be the case for IT-supported prehabilitation, as the consequences of ineffective preoperative risk reduction have tremendous negative effects on individual patients, their families, and society at large. Given the updated European guidelines for medical devices to include apps in higher risk classification, hopefully, more IT solutions will be investigated properly.

In conclusion, it seems doable to pilot the prototype of apps in a hierarchical and randomized design and to improve the functionality based on the comments from the users, both patients and staff. The next steps are to run randomized controlled trials on the effect on lifestyle and postoperative complications before possible implementation in the surgical setting.

## Data Availability

The data and material are not available due to the signed information by the participants regarding the use and the GDPR.

## References

[1] Eliasen M, Grønkjær M, Skov-Ettrup LS, Mikkelsen SS, Becker U, Tolstrup JS, Flensborg-Madsen T (2013). Preoperative alcohol consumption and postoperative complications: a systematic review and meta-analysis. Ann Surg.

[2] Grønkjær M, Eliasen M, Skov-Ettrup LS, Tolstrup JS, Christiansen AH, Mikkelsen SS, Becker U, Flensborg-Madsen T (2014). Preoperative smoking status and postoperative complications: a systematic review and meta-analysis. Ann Surg.

[3] ASA Physical Status Classification System. https://www.asahq.org/standards-and-guidelines/asa-physical-status-classification-system. Accessed 28 Sep 2021

[4] Durand F, Berthelot P, Cazorla C (2013). Smoking is a risk factor of organ/space surgical site infection in orthopaedic surgery with implant materials. Int Orthop.

[5] Agrawal S, Ingrande J, Said ET, Gabriel RA (2021). The association of preoperative smoking with postoperative outcomes in patients undergoing total hip arthroplasty. J Arthroplast.

[6] Tønnesen H, Nielsen PR, Lauritzen JB, Møller A (2009). Smoking and alcohol intervention before surgery: evidence for best practice. Br J Anaesth.

[7] Tønnesen H, Raffing R, Svane JK, Lauritzen JB, Thind PO, Lauridsen SV, Wernerman SO, Fagerlund MJ, Wiksell R, Berman AH, Combalia A, Lozano L, Fernández-Valencia JA, Santina M, Adami J, Spies C (2017) Lifestyle Intervention in the perioperative process – prior to digital intervention: Clinical Evidence and Knowledge Syntheses. Report 7.1 (H2020, grant 727558 Work Package 7)

[8] Thomsen T, Villebro N, Møller A (2014) Interventions for preoperative smoking cessation. Cochrane Database Syst Rev. 10.1002/14651858.CD002294.pub4 10.1002/14651858.CD002294.pub4 PMC7138216 24671929

[9] Egholm JWM, Pedersen B, Møller AM, Adami J, Juhl CB, Tønnesen H (2018) Perioperative alcohol cessation intervention for postoperative complications. Cochrane Database Syst Rev. 10.1002/14651858.CD008343.pub3 10.1002/14651858.CD008343.pub3 PMC6517044 30408162

[10] Pei H, Zhang LJ, Zeng LM, Yu F (2014). Effect of preoperative smoking intervention on postoperative complications of total hip replacement. Chinese J Evidence-Based Med.

[11] Lindström D, Azodi OS, Wladis A, Tønnesen H, Linder S, Nåsell H, Ponzer S, Adami J (2008). Effects of a Perioperative smoking cessation intervention on postoperative complications a randomized trial. Ann Surg.

[12] Møller AM, Villebro N, Pedersen T, Tønnesen H (2002). Effect of preoperative smoking intervention on postoperative complications: a randomised clinical trial. Lancet.

[13] Wong J, An D, Urman RD, Warner DO, Tønnesen H, Raveendran R, Abdullah HR, Pfeifer K, Maa J, Finegan B, Li E, Webb A, Edwards AF, Preston P, Bentov N, Richman D (2020). Society for perioperative assessment and quality improvement (SPAQI) consensus statement on perioperative smoking cessation. Anesth Analg.

[14] Svane JK, Chiou ST, Groene O, Kalvachova M, Brkić MZ, Fukuba I, Härm T, Farkas J, Ang Y, Andersen MØ, Tønnesen H (2018). A WHO-HPH operational program versus usual routines forimplementing clinical health promotion: an RCT in health promoting hospitals (HPH). Implement Sci.

[15] Åsberg K, Bendtsen M (2021). Perioperative digital behaviour change interventions for reducing alcohol consumption, improving dietary intake, increasing physical activity and smoking cessation: a scoping review. Perioper Med.

[16] Lemanu DP, Singh PP, Shao RY, Pollock TT, MacCormick AD, Arroll B, Hill A (2018). Text messaging improves preoperative exercise in patients undergoing bariatric surgery. ANZ J Surg.

[17] Landis JR, Koch GG (1977). The measurement of observer agreement for categorical data. Biometrics.

[18] Tobacco Use and Dependence Guideline Panel (2008). Treating tobacco use and dependence: 2008 update. Ann J Prev Med.

[19] Kulinski K, Smith N (2020). Surgical prehabilitation using mobile health coaching in patients with obesity: a pilot study. Anaesth Intensive Care.

[20] Mundi MS, Lorentz PA, Grothe K, Kellogg TA, Collazo-Clavell M (2015). No title feasibility of smartphone-based education modules and ecological momentary assessment/intervention in pre-bariatric surgery patients. Obes Surg.

[21] McCrabb S, Baker AL, Attia J, Balogh ZJ, Lott N, Naylor J, Harris IA, Doran CM, George J, Wolfenden L, Skelton E, Bonevski B (2017). Smoke-free recovery from trauma surgery : a pilot trial of an online smoking cessation program for orthopaedic trauma patients. Int J Environ Res Public Health.

[22] Thomas K, Bendtsen M, Linderoth C, Bendtsen P (2020). Implementing facilitated access to a text messaging, smoking cessation intervention among Swedish patients having elective surgery: qualitative study of patients’ and health care professionals’ perspectives. JMIR Mhealth Uhealth.

[23] Low CA, Danko M, Durica KC, Kunta AR, Ren Y, Bartlett DL, Bovbjerg D, Dey AK, John M (2020). A real-time mobile intervention to reduce sedentary behavior before and after cancer surgery : usability and feasibility study corresponding author. JMIR Perioper Med.

[24] Nolan M, Warner MA, Jacobs MA, Amato MS, Graham AL, Warner D (2019). Feasibility of a perioperative text messaging smoking cessation program for surgical patients. Anesth Analg.

[25] DeMartini KS, Schilsky ML, Palmer A, Fehon DC, Zimbrean P, O’Malley SS, Lee HB, Toll B (2018). Text messaging to reduce alcohol relapse in pre-listing liver transplant candidates: a pilot feasibility study. Alcohol Clin Exp Res.

[26] Krebs P, Burkhalter J, Fiske J, Snow H, Schofield E, Iocolano M, Borderud S, Ostroff J (2019) The QuitIT coping skills game for promoting tobacco cessation among smokers diagnosed with cancer: pilot randomized controlled trial. JMIR Mhealth Uhealth 7. 10.2196/10071 10.2196/10071 PMC6329892 30632971

[27] Velde MV, Valkenet K, Geleijn E, Kruisselbrink M, Marsman M, Janssen LMJ, Ruurda JP, Peet DLV, Aarden JJ, Veenhof C, Leeden M (2021) Usability and preliminary effectiveness of a preoperative mHealth app for people undergoing major surgery: pilot randomized controlled trial. JMIR Mhealth Uhealth 9. 10.2196/23402 10.2196/23402 PMC7819776 33410758

